# Supplementary material for: Determining seropositivity—A review of approaches to define population seroprevalence when using multiplex bead assays to assess burden of tropical diseases
Source: PLoS Negl Trop Dis. 2021 Jun 28;15(6):e0009457. doi: 10.1371/journal.pntd.0009457 (PMC8270565; doi:10.1371/journal.pntd.0009457)
Supplement: S1 Table — (DOCX) [file pntd.0009457.s001.docx]

**Supplementary Table 1. List of Articles Reviewed**. Reviewed articles with antigens within the study, study populations, and applied methods (indicated with X). ROC= receiver operating characteristic curve, PU= Presumed Unexposed, MM= Mixture Model, IS= International Standards, VIP= Visual Inflection Point, Q= Quantile (antigen titres), PEC= Pre-exposed Endemic Cohort, CPR=Cut-off previously reported (not included as a cut-off approach). The total number for each method is included in the bottom row.

| **Article** | **Antigen(s)** | **Population** | **ROC** | **PU** | **MM** | **IS** | **VIP** | **Q** | **PEC** | **CPR** |
| --- | --- | --- | --- | --- | --- | --- | --- | --- | --- | --- |
| ARNOLD et al. 2019 [1] | *Giardia* VSP3 + VSP5, *Cryptosporidium* Cp17 + Cp23, *E. histolytica* LecA, *Salmonella* LPS grp B + grp D, ETEC LT B, *Norovirus* GI.4 + GII. 4.NO, Cholera toxin B, *Campylobacter* p18, p39 | Haiti  Kenya  Tanzania | X |  | X |  |  |  | X |  |
| ARNOLD et al. 2014 [2] | *P. falciparum* MSP1_19_ | Haiti |  | X |  |  |  |  |  |  |
| ARNOLD et al. 2017 [3] | Wb123 antigen *P*. *falciparum* antibody response measured with the IgG indirect fluorescent antibody (IFA) test, *Cryptosporidium parvum* Cp17 and Cp23, *Escherichia coli* (ETEC) heat-labile toxin β subunit (EtxB), *E. histolytica* Gal/GalNAc lectin heavy chain subunit (LecA), lipopolysaccharide (LPS) from Salmonella enterica serotype Typhimurium (Group B), Purified recombinant norovirus GI.4 and GII.4 | Cook Islands  Nigeria  Haiti  United States |  |  | X |  |  |  |  |  |
| ASSEFA et al. 2019 [4] | six *Plasmodium* antigens: four human malaria species-specific merozoite surface protein-1 19kD antigens (MSP-1) and Apical Membrane Antigen-1 (AMA-1) for *Plasmodium falciparum* and *Plasmodium vivax*. | Ethiopia |  |  | X |  |  |  |  |  |
| AUGUSTINE et al. 2017 [5] | *Campylobacter jejuni* Heat-killed whole bacterial cells, *Helicobacter pylori* Bacterial cell lysate, *Toxoplasma gondii* SAG1, *Hepatitis A virus* Cell culture concentrate, Norovirus GI.1, Norovirus GII.4. | Puerto Rico |  | X |  |  |  |  |  |  |
| BONI et al. 2013 [6] | Influenza HA 1 | Vietnam |  |  |  |  |  | X |  |  |
| CHARD et al. 2018 [7] | *Campylobacter jejuni* p18, p39 *Cryptosporidium parvum* 17 kda 27 kda*,* dengue 2, dengue 3, *Entamoeba histolytica, Escherichia coli, Giardia intestinalis (VSP 3, VSP 5)* *Norovirus (Sydney strain), Plasmodium falciparum (MSP19, 42, AMA1) Salmonella enteritidis,* *Salmonella typhimurium, Schistosoma mansoni, Chlamydia trachomatis CT-694, Pgp, Vibrio cholerae* | Mali |  |  |  |  |  |  |  | X |
| CHU et al. 2013 [8] | ICT test for filarial antigens, BmR1 | American Samoa, Burkina Faso, Dominican Republic, Ghana, Indonesia, Malaysia, Philippines, Sri Lanka, Tanzania, Togo, and Vanuatu |  |  |  | X |  |  |  |  |
| COOLEY et al. 2016 [9] | *Treponema pallidum* recombinant rp 1 and treponemal membrane antibody TmpA | Ghana  Papua New Guinea | X |  |  |  |  |  |  |  |
| DEL FAVA et al. 2016 [10] | VZ Virus | Norway |  |  | X |  |  |  |  |  |
| FEESER et al. 2017 [11] | Onchocerca volvulus OV-16, OV-17, and OV-33, other filarial antigens | Uganda  Ethiopia | X |  |  |  |  |  |  |  |
| FILOMENA et al. 2017 [12] | HAV- VP4-VP2, VP3, and VP1, HBV- HBcAg HBsAg ad and HBsAg ay, HCV - Core g4a, Core g1b, NS3 g1a, and NS3 g1b, CMV- whole cell lysate of strain AD169, T. gondii whole tachyzoites, *H. pylori*  soluble protein extract of strain 49503 | European |  | X |  |  |  |  |  |  |
| FUJII et al. 2014 [13] | *Entamoeba histolytica* (C-IgL), *Leishmania donovani* (KRP42), *Toxoplasma gondii* (SAG1), *Wuchereria bancrofti* (SXP1), HIV (gag, gp120 and gp41), and *Vibrio cholerae* (cholera toxin) | Kenya |  | X | X |  |  |  |  |  |
| GOODHEW et al. 2014 [14] | *Chlamydia trachomatis* Pgp3 and CT694 | Tanzania | X |  |  |  |  |  |  |  |
| GOODHEW et al. 2012 [15] | *Chlamydia trachomatis* Pgp3 and CT694 | Tanzania |  | X |  |  |  |  |  |  |
| GWYN et al. 2017 [16] | *Chlamydia trachomatis* Pgp3 | Tanzania | X |  |  |  |  |  |  |  |
| HAMLIN et al. 2012 [17] | W. bancrofti Wb123, Brugia malayi Bm33 and Bm14, WSP from Wolbachia | Haiti |  | X |  |  |  |  |  |  |
| HARDELID et al. 2008 [18] | Rubella IgG | United Kingdom |  |  | X |  |  |  |  |  |
| KAAIJK et al. 2015 [19] | Anti-mumps nucleoprotein antibody | Netherlands |  |  |  |  |  |  |  | X |
| LIU et al. 2012 [20] | rotavirus A (RVA), noroviruses (NoVs) (including genogroups GI and GII), sapoviruses (SaV), human astrovirus (HAstV), enteric adenoviruses (EAds), and human bocavirus 2 (HBoV2) | China |  | X |  |  |  |  |  |  |
| MIGCHELSEN et al. 2017 [21] | *Chlamydia trachomatis* Pgp3 | Laos  Uganda  Gambia |  | X |  |  | X |  |  |  |
| MOSS et al. 2017 [22] | *W. bancrofti* Wb123, *Brugia malayi* Bm14 | Mali |  | X |  |  |  |  |  |  |
| MOSS et al. 2011 [23] | *Brugia malayi* Bm33 and Bm14 | Haiti |  | X |  |  |  |  |  |  |
| MOSS et al. 2014 [24] | *E. histolytica* LecA , *C.parvum*, Cry17, and Cry27, *G. intestinalis* assemblage A, VSP1–VSP3, and two from assemblage B, VSP4 and VSP5 | Haiti |  | X |  |  |  |  |  |  |
| NJENGA et al. 2020 [25] | *Brugia malayi* Bm33 and Bm14, *Strongyloides stercoralis* NIE, *P. falciparum* MSP1_19_, *W. bancrofti* Wb123, *Schistosoma mansoni* SEA + GST, Tetantus toxoid, diphtheria toxoid, measles virus neutralizing antibody | Kenya | X | X | X | X |  |  |  |  |
| ONDIGO et al. 2018 [26] | *P. falciparum* MSP1_19_*, Schistosoma mansoni* SEA, *Strongyloides stercoralis* NIE, diphtheria toxoid, Tetanus toxoid, Measles nucleoprotein, *Ascaris suum* AsHb | Kenya | X |  | X | X |  |  |  |  |
| PLUCINSKI et al. 2018 [27] | *P. falciparum* MSP1_19_ *+* CSP + LSA, *W. bancrofti* Wb123, *B. malayi Bm33, Strongyloides stercoralis* NIE | Mozambique | X | X |  |  |  |  |  |  |
| POIRIER et al 2016 [28] | Recombinant chikungunya virus antigen, dengue DENV-2 and DENV-3, P. falciparum antigens 19-kDa fragment, from clone 3D7, linked to glutathione-S-transferase, 42-kDa fragment from clone 3D7, 42-kDa fragment from clone FVO | Haiti |  | X |  |  |  |  |  |  |
| PRIEST et al. 2016 [29] | NIE for *Strongyloides stercoralis,*  SAG2A for *Toxoplasma gondii*, T24H for cysticercosis, PfMSP-1_19_ (3D7 strain) and PfMSP-1_42_ (3D7 strain and FVO strain) for *P*. *falciparum* malaria, PvMSP-1_19_ (Belem strain) for *P*. *vivax* malaria,  For lymphatic filariasis, *Brugia malayi* Bm14 and *W*. *bancrofti* Wb12 | Cambodia |  | X |  |  |  |  |  |  |
| PRIEST et al. 2015 [30] | Toxoplasma SAG2A | Haiti | X |  |  |  |  |  |  |  |
| RASCOE et al. 2015 [31] | NIE for *Strongyloides stercoralis* | Haiti |  | X |  |  |  |  |  |  |
| REDER et al. 2008 [32] | tetanus toxin, diphtheria toxin, and pertussis toxin | Germany |  |  |  | X |  |  |  |  |
| ROGIER et al. 2015 [33] | 42kD fragment of MSP-1: MSP-1p42(D) and MSP-1p42(F) from the 3D7 and FVO strains, respectively, 19 kD fragment (MSP-1p19) fused to glutathione S-transferase (GST) cloned from *P. falciparum* isolate 3D7. | Haiti |  | X | X |  |  |  |  |  |
| ROGIER et al. 2018 [34] | *Chikungunya* Anti-CHIKV IgG | Haiti |  | X |  |  |  |  |  |  |
| RONNBERG et al. 2017 [35] | whole virus antigens (WV), recombinant glycosylated E proteins (E), and non-structural protein 1 (NS1) | European Travelers to endemic countries |  | X |  |  |  |  |  |  |
| SCOBIE et al. 2016 [36] | Tetanus toxoid | Cambodia |  |  |  | X |  |  |  |  |
| SCOBIE et al. 2017 [37] | Tetanus toxoid | Kenya  Tanzania  Mozambique |  |  |  | X |  |  |  |  |
| SEPULVEDA et al. 2015 [38] | *P. falciparum* MSP 1 and AMA | African/ Non African |  |  | X |  |  |  |  |  |
| SEPULVEDA et al. 2015 [39] | *P. falciparum* MSP 1 and AMA | Equatorial Guinea |  |  | X |  |  |  |  |  |
| VYSE et al. 2006 [40] | Rubella. measles and mumps-specific IgG | England  Wales |  |  | X |  |  |  |  |  |
| WEST et al. 2018 [41] | *Chlamydia trachomatis* Pgp3 | Tanzania | X |  |  |  |  |  |  |  |
| WIEGAND et al. 2018 [42] | *Chlamydia trachomatis* Pgp3 | Bolivia  Nepal  United States | X |  | X |  |  |  |  |  |
| WILSON et al. 2016 [43] | *O*. *volvulus* Ov16 *W. bancrofti* Wb123 | Senegal |  |  |  |  |  |  |  | X |
| WON et al. 2017 [44] | *Schistosoma mansoni* SEA and Sm25 | Kenya | X | X |  |  |  |  |  |  |
| WON et al. 2018 [45] | *W. bancrofti* Wb123, *Brugia malayi* Bm33 and Bm14 | American Samoa | X | X |  |  |  |  |  |  |
| WON et al. 2018 [46] | *W. bancrofti* Wb123, *Brugia malayi* Bm14 | Gambia | X | X |  |  |  |  |  |  |
| ZAMBRANO et al. 2017 [47] | *Cryptosporidium parvum* Cp17 and Cp23, *Escherichia coli* (ETEC) heat-labile toxin β subunit (EtxB), *E. histolytica* Gal/GalNAc lectin heavy chain subunit (LecA), *T. gondii* SAG2A | Rwanda | X | X | X |  |  |  |  |  |
| Total number |  |  | 15 | 23 | 14 | 6 | 1 | 1 | 1 | 3 |

1. Arnold BF, Martin DL, Juma J, Mkocha H, Ochieng JB, Cooley GM, et al. Enteropathogen antibody dynamics and force of infection among children in low-resource settings. Elife. 2019;8.

2. Arnold BF, Priest JW, Hamlin KL, Moss DM, Colford JM, Jr., Lammie PJ. Serological measures of malaria transmission in Haiti: comparison of longitudinal and cross-sectional methods. PLoS One. 2014;9(4):e93684.

3. Arnold BF, van der Laan MJ, Hubbard AE, Steel C, Kubofcik J, Hamlin KL, et al. Measuring changes in transmission of neglected tropical diseases, malaria, and enteric pathogens from quantitative antibody levels. PLoS Negl Trop Dis. 2017;11(5):e0005616.

4. Assefa A, Ali Ahmed A, Deressa W, Sime H, Mohammed H, Kebede A, et al. Multiplex serology demonstrate cumulative prevalence and spatial distribution of malaria in Ethiopia. Malar J. 2019;18(1):246.

5. Augustine SAJ, Simmons KJ, Eason TN, Curioso CL, Griffin SM, Wade TJ, et al. Immunoprevalence to Six Waterborne Pathogens in Beachgoers at Boqueron Beach, Puerto Rico: Application of a Microsphere-Based Salivary Antibody Multiplex Immunoassay. Front Public Health. 2017;5:84.

6. Boni MF, Chau NV, Dong N, Todd S, Nhat NT, de Bruin E, et al. Population-level antibody estimates to novel influenza A/H7N9. J Infect Dis. 2013;208(4):554-8.

7. Chard AN, Trinies V, Moss DM, Chang HH, Doumbia S, Lammie PJ, et al. The impact of school water, sanitation, and hygiene improvements on infectious disease using serum antibody detection. PLoS Negl Trop Dis. 2018;12(4):e0006418.

8. Chu BK, Deming M, Biritwum NK, Bougma WR, Dorkenoo AM, El-Setouhy M, et al. Transmission assessment surveys (TAS) to define endpoints for lymphatic filariasis mass drug administration: a multicenter evaluation. PLoS Negl Trop Dis. 2013;7(12):e2584.

9. Cooley GM, Mitja O, Goodhew B, Pillay A, Lammie PJ, Castro A, et al. Evaluation of Multiplex-Based Antibody Testing for Use in Large-Scale Surveillance for Yaws: a Comparative Study. J Clin Microbiol. 2016;54(5):1321-5.

10. Del Fava E, Rimseliene G, Flem E, Freiesleben de Blasio B, Scalia Tomba G, Manfredi P. Estimating Age-Specific Immunity and Force of Infection of Varicella Zoster Virus in Norway Using Mixture Models. PLoS One. 2016;11(9):e0163636.

11. Feeser KR, Cama V, Priest JW, Thiele EA, Wiegand RE, Lakwo T, et al. Characterizing Reactivity to Onchocerca volvulus Antigens in Multiplex Bead Assays. Am J Trop Med Hyg. 2017;97(3):666-72.

12. Filomena A, Pessler F, Akmatov MK, Krause G, Duffy D, Gartner B, et al. Development of a Bead-Based Multiplex Assay for the Analysis of the Serological Response against the Six Pathogens HAV, HBV, HCV, CMV, T. gondii, and H. pylori. High Throughput. 2017;6(4).

13. Fujii Y, Kaneko S, Nzou SM, Mwau M, Njenga SM, Tanigawa C, et al. Serological surveillance development for tropical infectious diseases using simultaneous microsphere-based multiplex assays and finite mixture models. PLoS Negl Trop Dis. 2014;8(7):e3040.

14. Goodhew EB, Morgan SM, Switzer AJ, Munoz B, Dize L, Gaydos C, et al. Longitudinal analysis of antibody responses to trachoma antigens before and after mass drug administration. BMC Infect Dis. 2014;14:216.

15. Goodhew EB, Priest JW, Moss DM, Zhong G, Munoz B, Mkocha H, et al. CT694 and pgp3 as serological tools for monitoring trachoma programs. PLoS Negl Trop Dis. 2012;6(11):e1873.

16. Gwyn S, Cooley G, Goodhew B, Kohlhoff S, Banniettis N, Wiegand R, et al. Comparison of Platforms for Testing Antibody Responses against the Chlamydia trachomatis Antigen Pgp3. Am J Trop Med Hyg. 2017;97(6):1662-8.

17. Hamlin KL, Moss DM, Priest JW, Roberts J, Kubofcik J, Gass K, et al. Longitudinal monitoring of the development of antifilarial antibodies and acquisition of Wuchereria bancrofti in a highly endemic area of Haiti. PLoS Negl Trop Dis. 2012;6(12):e1941.

18. Hardelid P, Williams D, Dezateux C, Tookey PA, Peckham CS, Cubitt WD, et al. Analysis of rubella antibody distribution from newborn dried blood spots using finite mixture models. Epidemiol Infect. 2008;136(12):1698-706.

19. Kaaijk P, Gouma S, Hulscher HI, Han WG, Kleijne DE, van Binnendijk RS, et al. Dynamics of the serologic response in vaccinated and unvaccinated mumps cases during an epidemic. Hum Vaccin Immunother. 2015;11(7):1754-61.

20. Liu Y, Xu ZQ, Zhang Q, Jin M, Yu JM, Li JS, et al. Simultaneous detection of seven enteric viruses associated with acute gastroenteritis by a multiplexed Luminex-based assay. J Clin Microbiol. 2012;50(7):2384-9.

21. Migchelsen SJ, Martin DL, Southisombath K, Turyaguma P, Heggen A, Rubangakene PP, et al. Defining Seropositivity Thresholds for Use in Trachoma Elimination Studies. PLoS Negl Trop Dis. 2017;11(1):e0005230.

22. Moss DM, Chard AN, Trinies V, Doumbia S, Freeman MC, Lammie PJ. Serological Responses to Filarial Antigens in Malian Children Attending Elementary Schools. Am J Trop Med Hyg. 2017;96(1):229-32.

23. Moss DM, Priest JW, Boyd A, Weinkopff T, Kucerova Z, Beach MJ, et al. Multiplex bead assay for serum samples from children in Haiti enrolled in a drug study for the treatment of lymphatic filariasis. Am J Trop Med Hyg. 2011;85(2):229-37.

24. Moss DM, Priest JW, Hamlin K, Derado G, Herbein J, Petri WA, Jr., et al. Longitudinal evaluation of enteric protozoa in Haitian children by stool exam and multiplex serologic assay. Am J Trop Med Hyg. 2014;90(4):653-60.

25. Njenga SM, Kanyi HM, Arnold BF, Matendechero SH, Onsongo JK, Won KY, et al. Integrated Cross-Sectional Multiplex Serosurveillance of IgG Antibody Responses to Parasitic Diseases and Vaccines in Coastal Kenya. Am J Trop Med Hyg. 2020;102(1):164-76.

26. Ondigo BN, Muok EMO, Oguso JK, Njenga SM, Kanyi HM, Ndombi EM, et al. Impact of Mothers' Schistosomiasis Status During Gestation on Children's IgG Antibody Responses to Routine Vaccines 2 Years Later and Anti-Schistosome and Anti-Malarial Responses by Neonates in Western Kenya. Front Immunol. 2018;9:1402.

27. Plucinski MM, Candrinho B, Chambe G, Muchanga J, Muguande O, Matsinhe G, et al. Multiplex serology for impact evaluation of bed net distribution on burden of lymphatic filariasis and four species of human malaria in northern Mozambique. PLoS Negl Trop Dis. 2018;12(2):e0006278.

28. Poirier MJ, Moss DM, Feeser KR, Streit TG, Chang GJ, Whitney M, et al. Measuring Haitian children's exposure to chikungunya, dengue and malaria. Bull World Health Organ. 2016;94(11):817-25A.

29. Priest JW, Jenks MH, Moss DM, Mao B, Buth S, Wannemuehler K, et al. Integration of Multiplex Bead Assays for Parasitic Diseases into a National, Population-Based Serosurvey of Women 1539 Years of Age in Cambodia. Plos Neglect Trop D. 2016;10(5).

30. Priest JW, Moss DM, Arnold BF, Hamlin K, Jones CC, Lammie PJ. Seroepidemiology of Toxoplasma in a coastal region of Haiti: multiplex bead assay detection of immunoglobulin G antibodies that recognize the SAG2A antigen. Epidemiol Infect. 2015;143(3):618-30.

31. Rascoe LN, Price C, Shin SH, McAuliffe I, Priest JW, Handali S. Development of Ss-NIE-1 recombinant antigen based assays for immunodiagnosis of strongyloidiasis. PLoS Negl Trop Dis. 2015;9(4):e0003694.

32. Reder S, Riffelmann M, Becker C, Wirsing von Konig CH. Measuring immunoglobulin g antibodies to tetanus toxin, diphtheria toxin, and pertussis toxin with single-antigen enzyme-linked immunosorbent assays and a bead-based multiplex assay. Clin Vaccine Immunol. 2008;15(5):744-9.

33. Rogier E, Wiegand R, Moss D, Priest J, Angov E, Dutta S, et al. Multiple comparisons analysis of serological data from an area of low Plasmodium falciparum transmission. Malar J. 2015;14:436.

34. Rogier EW, Moss DM, Mace KE, Chang M, Jean SE, Bullard SM, et al. Use of Bead-Based Serologic Assay to Evaluate Chikungunya Virus Epidemic, Haiti. Emerging Infectious Diseases. 2018;24(6):995-1001.

35. Ronnberg B, Gustafsson A, Vapalahti O, Emmerich P, Lundkvist A, Schmidt-Chanasit J, et al. Compensating for cross-reactions using avidity and computation in a suspension multiplex immunoassay for serotyping of Zika versus other flavivirus infections. Med Microbiol Immunol. 2017;206(5):383-401.

36. Scobie HM, Mao B, Buth S, Wannemuehler KA, Sorensen C, Kannarath C, et al. Tetanus Immunity among Women Aged 15 to 39 Years in Cambodia: a National Population-Based Serosurvey, 2012. Clin Vaccine Immunol. 2016;23(7):546-54.

37. Scobie HM, Patel M, Martin D, Mkocha H, Njenga SM, Odiere MR, et al. Tetanus Immunity Gaps in Children 5-14 Years and Men >/= 15 Years of Age Revealed by Integrated Disease Serosurveillance in Kenya, Tanzania, and Mozambique. Am J Trop Med Hyg. 2017;96(2):415-20.

38. Sepulveda N, Drakeley C. Sample size determination for estimating antibody seroconversion rate under stable malaria transmission intensity. Malar J. 2015;14:141.

39. Sepulveda N, Stresman G, White MT, Drakeley CJ. Current Mathematical Models for Analyzing Anti-Malarial Antibody Data with an Eye to Malaria Elimination and Eradication. J Immunol Res. 2015;2015:738030.

40. Vyse AJ, Gay NJ, Hesketh LM, Pebody R, Morgan-Capner P, Miller E. Interpreting serological surveys using mixture models: the seroepidemiology of measles, mumps and rubella in England and Wales at the beginning of the 21st century. Epidemiol Infect. 2006;134(6):1303-12.

41. West SK, Munoz B, Kaur H, Dize L, Mkocha H, Gaydos CA, et al. Longitudinal change in the serology of antibodies to Chlamydia trachomatis pgp3 in children residing in a trachoma area. Sci Rep. 2018;8(1):3520.

42. Wiegand RE, Cooley G, Goodhew B, Banniettis N, Kohlhoff S, Gwyn S, et al. Latent class modeling to compare testing platforms for detection of antibodies against the Chlamydia trachomatis antigen Pgp3. Sci Rep. 2018;8(1):4232.

43. Wilson NO, Badara Ly A, Cama VA, Cantey PT, Cohn D, Diawara L, et al. Evaluation of Lymphatic Filariasis and Onchocerciasis in Three Senegalese Districts Treated for Onchocerciasis with Ivermectin. PLoS Negl Trop Dis. 2016;10(12):e0005198.

44. Won KY, Kanyi HM, Mwende FM, Wiegand RE, Goodhew EB, Priest JW, et al. Multiplex Serologic Assessment of Schistosomiasis in Western Kenya: Antibody Responses in Preschool Aged Children as a Measure of Reduced Transmission. Am J Trop Med Hyg. 2017;96(6):1460-7.

45. Won KY, Robinson K, Hamlin KL, Tufa J, Seespesara M, Wiegand RE, et al. Comparison of antigen and antibody responses in repeat lymphatic filariasis transmission assessment surveys in American Samoa. Plos Neglect Trop D. 2018;12(3).

46. Won KY, Sambou S, Barry A, Robinson K, Jaye M, Sanneh B, et al. Use of Antibody Tools to Provide Serologic Evidence of Elimination of Lymphatic Filariasis in The Gambia. American Journal of Tropical Medicine and Hygiene. 2018;98(1):15-20.

47. Zambrano LD, Priest JW, Ivan E, Rusine J, Nagel C, Kirby M, et al. Use of Serologic Responses against Enteropathogens to Assess the Impact of a Point-of-Use Water Filter: A Randomized Controlled Trial in Western Province, Rwanda. American Journal of Tropical Medicine and Hygiene. 2017;97(3):876-87.
