# Supplementary material for: Determining seropositivity—A review of approaches to define population seroprevalence when using multiplex bead assays to assess burden of tropical diseases
Source: PLoS Negl Trop Dis. 2021 Jun 28;15(6):e0009457. doi: 10.1371/journal.pntd.0009457 (PMC8270565; doi:10.1371/journal.pntd.0009457)
Supplement: Attachment — Submitted filename: Chen et al 2020 - MN final.docx [file pntd.0009457.s002.docx]

**Determining seropositivity – A review of approaches to define seroprevalence when using multiplex bead assays to assess burden of tropical diseases**

**Review**

Suitable quality?: Yes

Sufficient general interest?: Yes

Conclusions justified?: Yes

Clearly written?: Yes

Procedures described?: Yes

Supplemental material warranted?: No

Comments:

In this manuscript, Chan et al. reviewed the literature surrounding the use of multiplex bead assays on human sera to assess the burden of neglected tropical diseases. The authors focus on the various cut-off methods that have been employed to define the seroprevalence of disease, covering the advantages and disadvantages of each major method. The authors then perform two case studies, applying three of the major cut-off methods to multiplex bead assay data on serological samples from Haiti and from Malaysia. The results of these case studies demonstrate that the interpreted seroprevalence of three tropical diseases (Filariasis, Strongyloidiasis and Trachoma) in these populations are largely dependent on the cut-off methodology that was employed. The results also demonstrate that different cut-off methodologies can affect the apparent spatial distribution of disease prevalence in a region, which the authors rightly discuss as a potential issue for decisions regarding disease control or elimination activities.

The manuscript is well written with exceptional organization and clarity of thought. The major issue that the authors address; serological cut-off methodology, is of critical importance for epidemiological research on the seroprevalence of tropical diseases as determined through multiplex bead assays (the focus of this study) as well as other serological methods to assess the disease burden of a population. The number of articles that were included in this review is relatively low (56), however this is not surprising due to the highly focussed inclusion criteria.

The case studies provide an excellent proof-of-concept, highlighting the variability between study cohorts and study parameters, and the importance of careful consideration of these variables in order to select the most appropriate cut-off methodology for each specific situation. The methodology is clear with I believe adequate details for the work to be reproduced by other researchers.

The authors finish the article by including detailed guidelines to consider when selecting cut-off methodology for serological investigations into disease prevalence. The work and ideas described in this manuscript are an excellent resource that can be used to improve the quality and reproducibility of epidemiological research. This study is a welcome addition to the scientific literature, and I would recommend it be accepted with some minor revisions.

There are minor issues in the manuscript as detailed below:

1. Title should be modified to reflect the 3 diseases (Filariasis, Strongyloidiasis and Trachoma: 2 helminths and 1 bacterial infection) discussed in the manuscript. When reading the title (….assess burden of tropical diseases), I had the impression more NTDs were listed in the manuscript.
2. In the abstract, lymphatic filariasis antigen targets are referred to as Bm14 and BM33. However, throughout the manuscript there is no mention of BM33 and instead the authors refer to antigens WB123 and BM14, which represent lymphatic filariasis. This discrepancy between the abstract and the manuscript must be rectified. Furthermore, the use of Bm14 and BM14 are used interchangeably. The authors should select one and remain consistent throughout the manuscript.
3. The authors assess the seroprevalence of lymphatic filariasis using two distinct antigens. However, lymphatic filariasis can be caused by more than a single filarial species, therefore a brief definition of the antigen’s source could provide some clarification.
4. The final sentence on page 7, line 141-142 appears to be incomplete and could be clarified.
5. In table 2, the authors use the term “unexposed or presumed negative population” for the first cut-off method. However, during the case study on page 16, line 322 and elsewhere, they refer to the method as “non-exposed”. The authors should select one and remain consistent throughout the manuscript.
6. The title of Table 3 on page 16, line 330-331 is unclear and incomplete. This should be re-written to more appropriately describe the table contents.
7. On page 20, line 408 the authors state “prevalence by household clusters were mapped in Malaysia Sabah for two antigens.” This is not demonstrated in Figure 3 which appears to focus on only one filarial antigen (Bm14). Perhaps the authors meant “for two cut-off methods” instead of antigens? This must be resolved.
