# Supplementary material for: Determining seropositivity—A review of approaches to define population seroprevalence when using multiplex bead assays to assess burden of tropical diseases
Source: PLoS Negl Trop Dis. 2021 Jun 28;15(6):e0009457. doi: 10.1371/journal.pntd.0009457 (PMC8270565; doi:10.1371/journal.pntd.0009457)
Supplement: Attachment — Submitted filename: ChanYYMBASeroReviewerComments.docx [file pntd.0009457.s003.docx]

**Manuscript PNTD-D-20-01828.**
Responses to Reviewers

Dear Reviewers,

Thank you kindly for providing comments and suggestions toward our manuscript. We have incorporated these suggestions into the manuscript and have provided a manuscript containing these tracked changes. Please see below, in red, for point-by-point response to reviewers comments and concerns.


**Reviewer #1:** The paper comprises two separate parts -a systematic review of cut-off determination methods, and a comparison of different methods used in Haiti and Malaysia. The two parts do not depend on one another or hang together particularly well.

Please consider separating this into two papers and then each part could be explored more fully.

Thank you for this feedback, the manuscript has been separated as recommended into two distinct papers. The first paper comprises of the review of literature while the second will focus on the case study.

The description of the search is confusing - how many more were found from the period between Oct 2018 and Jan 2020 and how many were from ref lists of first included studies? How many studies were in each method/category?

We have adjusted the description to clarify the search, including the timeframe we reviewed. The figure has also been updated to reflect this change. The number of studies in each method and category has been added in both Table 1 and Supplementary Table 1. [See pages 5 and 6 in revised manuscript]

Supp Table 1 is just a list of papers and Table 1 is very minimal - not clear how many did each method. Some locations that are in papers cited in Supp Table 1 do not appear in Table 1 (papers by Won in American Samoa and Gambia, for example). There may be others.

Thank you for this feedback. The purpose of the original Table 1 was to show examples of the application of MBAs and applied serological cut-offs in various settings and was not meant to be fully inclusive of all articles reviewed (full article list found in Supplementary Table 1, see page 19 in revised manuscript). The original Table 1 has now been modified to include examples of all methods reviewed and the number of articles that used each cut-off method. [See pages 10 and 11 in revised manuscript]

What cutoffs did the authors find in each paper with each antigen and method? DId it vary by transmission setting, technical factors or other?

Thank you for the suggestion. We have reorganized the Supplemental Table 1 by cut-off methods used in each paper, antigen, and source population. We did not compare outcome of cut-off methods by transmission settings or technical factors among the literature, however, this is included as an important consideration in Box 1 and within the discussion. The original inclusion of the case study was in fact to explore these considerations. [See page starting 19 in revised manuscript]

I am not sure what is gained from the papers to generate Table 2. It's a useful summary of potential biases, but could likely have been written without reading any of them.

We have decided to keep this as it is a helpful summary of advantageous and disadvantageous that may not be readily apparent to everyone, especially methods that may be unfamiliar or less frequently used.

Box 1 is very long and diverts into a lot of side topics. It may be useful but doesn't seem to arise from the data in this paper. Again could be valuable in separate review paper, but is strangely ordered after the case studies.

Thank you for this advice. Box 1 no longer appears after the case studies (case studies have been removed). The topics within the Box 1 have also been written into the discussion, with Box 1 references at the end of discussion. [See page 17 in revised manuscript]

The Discussions states some fairly obvious points - e.g. 440-441, lines 458 to 463 that could have been written before this paper and perhaps belong in introduction. We do not need this paper to tell us that cutoffs are important. How does this paper take us further? While the caveats in lines 445 to 447 are true, this is always the case.

The conclusion needs to be rewritten. Currently it is general, states the obvious and could have been written before the study was done. It does not draw any conclusion from the results, such as how many methods were found, or that there were site-specific differences even with the same method. No clear recommendation is made.

Thank you for the feedback. The conclusion has been revised based on suggested comments. The number of methods found was previously mentioned in beginning of the results section and is now included in the discussion with emphasis on more commonly used approaches. Additional recommendations for ideal international standards moving forward are discussed in further detail and provisional recommendations on which cut-off methods to use and why have been added in the discussion. [See pages 14- 17 in revised manuscript]

Don't be afraid to draw preliminary conclusions from the findings and make provisional recommendations.

Thank you, preliminary conclusions from review of literature regarding recommendations for appropriate cut-off method has been added in the discussion. [See page 16 in revised manuscript]

Title - I think it should specify Vaccine Preventable and Neglected Tropical Diseases. 'Tropical Diseases' is too broad. I was expecting malaria to be included.

We agree and this change has been made.

In the systematic review part, please give more details rather than just a list of who did what method. Make sure list is comprehensive. What cutoffs did they each find? What were the background transmission situations.? What stage was the programme at? Are these antigens/antibodies currently used in decision making? Should they be, now that MBA assays are available? Also, how about comparing with or at least mentioning studies that used mixture models with conventional ELISAs.

Thank you for these suggestions. These are important points, several which were previously included in Box 1. Supplementary Table 1 has been revised to include which papers used which method, the antigens in the study, and origins of the study populations. Direct comparisons of study-specific cut-offs in literature may not be as informative due to a variety of reasons such as population genetics, transmission settings, temporal variation, and laboratory methods. If the reason to dive into these factors is to provide a guide by which people can refer to, we believe factors in Box 1 would be more informative in considering method choice. Several of these studies have also used conventional ELISA in addition to MBAs

**Reviewer #2**: 1. A key concern is the cohesion in the paper between the review and the case study. A recommended solution would to consider segregating this into two separate publications or revise the case study with more focus on a couple of diseases as a an example.

2. It is unclear why the authors are emphasizing multiplex bead-based serology assays as a theme of the paper. The initial landscape section regarding analysis approaches could be adapted and would likely be applicable to methods used for defining cut-offs for quantitative serology assays regardless of platform. While there are certain characteristics that are specific to bead-based serology assays (such as read-out using MFI), wouldn't the issue of using different methods for defining cut-offs also be applicable to other quantitative serology platforms some of which have also been used to develop multiplex serology assays. Broadening the focus would likely add value as a more comprehensive review article with broader interest to people conducting seroepidemiology studies.

Thank you for this feedback. The initial interest of this manuscript arose due to the novelty of applying multiplex bead assays to integrated disease surveillance, where serology was not traditionally used for certain NTDs to inform programmatic decision making. The purpose was to highlight, for programs transitioning to using MBAs for integrated surveillance, the different methods for cut-off determination among antigens and subsequent implications. It is true that the issue with cut-off method does apply to other serological platforms using cut-offs as several studies reviewed also used ELISA platforms, which is discussed as a potential limitation. [See page 16 in revised manuscript]

**Reviewer #3**

Title should be modified to reflect the 3 diseases (Filariasis, Strongyloidiasis and Trachoma: 2 helminths and 1 bacterial infection) discussed in the manuscript. When reading the title (…. assess burden of tropical diseases), I had the impression more NTDs were listed in the manuscript.

Thank you for the suggestion. For the literature review, the title has been changed to neglected and vaccine preventable diseases to be more inclusive for MBAs. This suggestion will be included for the case study to reflect diseases examined.

In the abstract, lymphatic filariasis antigen targets are referred to as Bm14 and BM33. However, throughout the manuscript there is no mention of BM33 and instead the authors refer to antigens WB123 and BM14, which represent lymphatic filariasis. This discrepancy between the abstract and the manuscript must be rectified. Furthermore, the use of Bm14 and BM14 are used interchangeably. The authors should select one and remain consistent throughout the manuscript.

Thank you for the feedback. Bm33 has been removed, and Bm14 has been changed to be consistent throughout the manuscript.

In table 2, the authors use the term “unexposed or presumed negative population” for the first cut-off method. However, during the case study on page 16, line 322 and elsewhere, they refer to the method as “non-exposed”. The authors should select one and remain consistent throughout the manuscript.

Thank you for this advice. The reference to this term has been changed to “presumed negative” to be consistent throughout the paper to avoid any confusion.
