# Supplementary material for: Determining seropositivity—A review of approaches to define population seroprevalence when using multiplex bead assays to assess burden of tropical diseases
Source: PLoS Negl Trop Dis. 2021 Jun 28;15(6):e0009457. doi: 10.1371/journal.pntd.0009457 (PMC8270565; doi:10.1371/journal.pntd.0009457)
Supplement: Attachment — Submitted filename: ChanY_ReviewerCommentsandResponses.docx [file pntd.0009457.s004.docx]

**Manuscript PNTD-D-20-01828.**
Responses to Reviewers

Dear Reviewers,

Thank you very much for providing comments and suggestions toward our revised manuscript. We have made revisions based on these suggestions and have provided a manuscript containing these tracked changes. Please see below, in red, for point-by-point response to reviewers’ comments and concerns.

**Methods**

Reviewer #3: Line 108-110: the authors said that "Fifty five articles met the inclusion criteria for full
screening which included serology, NTDs (as defined by the World Health Organization)(15), malaria,
VPDs in tropical regions, and the use of MBA (Figure 1). However, they mentioned others pathogens not listed in WHO list. For TABLE 1: The authors have to choose between the NTDs recognized by WHO and NTDs recognized by PLoS NTD (see Hotez et al. PLoS NTD 2020 Jan 30;14(1):e0008001.
E.g Giardia, Entamoeba, ..etc

Thank you for this feedback. PloS NTD, while originally cited, has now been added to the text. We included the definition both by WHO and PLoS NTD.

**Results**

Reviewer #1: The tables in main document are fine. Table 1 should be placed earlier when it is first mentioned Page 6. The different cutoff determination methods are more clearly described.
Supplementary Table 1 - the cutoff determination methods are not in same order as in the text, and many papers are repeated. I believe Supplementary Table 1 would be much more useful if revised as follows:
List each paper only once and put it in short form e.g. (ref no) Arnold et al 2019. Provide additional columns at right to show which methods the paper uses (Presumed negative, ROC etc) with tick or Y in the column if they used relevant method. It will then be easier to see which papers used which methods, and which tested multiple methods. Most (all?) papers are referenced in main text or can be added so you can use the same number as reference. Or have a list of refs to supp material.
Then you can sum the number of papers using each method in last row.

Table 1 has now been moved to page 6.

Supplemental Table 1 has been adjusted according to these suggestions:

Papers are listed in short form and columns have been added for the different methods.

X indicates when a relevant method was applied.

References have been added to each paper in the supplemental table, and shares references within the main text.

The Finite Mixture model method is described incorrectly. Lines 168 to 171. It does not use mean and distribution of the 'negative population' since the composition of the negative population is not known. It postulates two or more distinct sub-populations (components) and assesses when probability of a data point/sample being in one or the other is >50% (or other defined value). The text in those lines seems to refer to the Presumed negative population method. Same problem in Table 1 for this method. It is not Mean + 3 SD. The component populations may overlap.

The mixture model description has been adjusted to clarify that the lowest component is presumed to be a seronegative population.

There has been an additional change to the text to clarify that cut-offs are determined using the lowest components and estimated parameters (e.g. mean or SD) identified by the mixture model, not the mixture model itself.

A sentence describing using mixture models and probability classification to determine cut-off values has also been added to the text.

Reviewer #3: Line: 182-184: I could not understand how would this model be able to differentiate between the naturally infected and the vaccinated individuals, is based on the cut-offs as discussed before or comparing to the nature of responses seen during the studies. Because the responses of the population might change due to various factors involved

A clarification has been added explain that different components used in mixture models can reflect titres of antibodies responses that have been found to indicate natural infection compared to vaccinated induced responses (as described in the literature).

TABLE 2. should be revised and linked with the references used in the text describing each cut-off method. Otherwise it is very difficult to follow. For example, for the International Standards, the reference # 32 is cited in the disadvantages but not cited in the text.

Thank you for the feedback, these two references have been removed to avoid confusion. We believe the point of this table serves as a summary of the main points discussed within each method section with appropriate references.

**Discussion**

Reviewer #1: Conclusions are reasonable. Please explain 'past precedents'. It is not clear to me.
There is a paragraph on limitations but may have missed a few - e.g. only papers in PubMed, only in English? Time boundaries?

These suggested limitations have been added into the discussion.

Clarification has been included within the text. Past precedents in this context means using cut-off approaches applied previously for an antigen of biologically similar diseases. These details have also been included within Box 1.

Reviewer #3: DISCUSSION:
Line 382: Would you expect any changes that would take place in the cut-offs for the various detection of parasites when they are couped together?

Thank you for the comment, this point has been added to the discussion. It is likely that cross-reactivity remains a challenge for multiplex assays. Well defined antigens are needed to avoid non-specific binding that could lead to inaccurate values.

Reviewer #4: The conclusions are supported by the data presented and the authors have acknowledged the limitations of their study design

--------------------

**Editorial and Data Presentation Modifications**

Reviewer #1: 1. Apparently malaria IS included (and is not in the categories of Vaccine Preventable (not quite yet) or NTDs. So perhaps revernt to using Tropical Diseases or alternatively just just delete the part of title after Mulitplex Bead Assays The title has been changed to Tropical Diseases
2. Explain past precedents line 36 (what was done before?) Explanation included for biologically similar antigens used previously or international guides.
3. line 78 - can you give a citation for use in evaluating MDA programs? Citation added
4. Table 1 for VIP-Trachoma - what do you mean by Non-impartial? Partial? Definition added to clarify as ‘independent’ individuals
5. Line 238. Maybe a word missing like 'and' before accuracy? And has been added
6. Lines 245-247. WHy would this method allow estimation of cutoffs for particularly high transmission pathogens where other methods failed? Can you elaborate and/or give an example? Examples and corresponding references have been added accordingly.
7. Lines 255 to 256. Wouldn't it be maternal immunity (transfer of antibody from mother to child) that is also a reason not to use infants as 'unexposed' individuals? Yes, this point has been included
8. Discussion Para 2 is very long and covers several topics. Please split into separate paragraphs. E.g. line 286 and 292 This has been separated into two paragraphs
9. Lines 323 - not clear why monoclonal antibodies are mentioned. To test for true positives and negs?

Yes, to test for true positives and negatives of specific antigens. To avoid repetition, this has been removed, as control sera has been previously defined.

10. In Box 1. Last para but one. Predominantly not predominately This has been corrected
11. Ref 64 word missing in title after Their. Added the missing world in the reference title

Reviewer #3: ABSTRACT:
Line 29: neglected tropical diseases or vaccine preventable diseases should be abbreviated, NTD and VPD, respectively.

The abbreviations have been added

**INTRODUCTION:**
Line 71-73: Some individuals might receive treatment for a specific disease and be still antibody positive for a while. How to distinguish dissociate this category and the asymptomatic group. In addition, since most of these NTD are present in developing and Lower-middle-income Countries a non-negligible of people self-medicate themselves.

Line 87-89: So when it is said that all these factors are combined and put under the umbrella of “seropositive”, but they also differ on the basis of the ½ life of antibodies, a little more explanation on how they would be affected would make his more understandable as it seems like a general statement. In addition, it is also good to mention how cross-reactivity would play a significant role when dressing these responses.

Thank you for the comments- this is a challenge to interpreting serological data. Prior knowledge of the specific antibody titres and associated kinetics would be helpful in more accurately interpreting the data. For example, it would be ideal if there were specific antibody signatures of recent treatment, asymptomatic infections, use of drugs etc., that would enable a more nuanced interpretation of serological results. Furthermore, specific antibody markers may provide detail toward infection or exposure history. Text has been added to the definition of seropositive for the purpose of this paper, and the point regarding issues of cross-reactivity is now included in the discussion.

**RESULTS:**
Line 136: "Any result above that MFI-bg value is considered as seropositive" should read ....above that MFI-bg plus 3 times the value of the standard deviation is considered....

This has been rearranged to follow the description of the method so it is clear.

**Line 148-150: When dealing with cross reactivity, the authors should elaborate and take in account people displaying polyclonal hypergammaglobulinaemia.**

We have highlighted in the discussion potential cross reactivity when using multiplex assays, and the necessity of well-defined antigens to avoid cross-reactivity and non-specific binding. Additionally, we have added a description regarding heterogeneity of individual responses that may not be due to pathogen exposure exclusively but can be influenced by other factors such as nutrition status and underlying health conditions. This can cause elevated antibodies such as hypergammaglobulinemia.

**REFERENCES:**

Line 381 and 384: be consistent. This has been corrected
Line 433: is missing Journal name and volume. The name of the Journal has been added
Line 515: Reference of a book Chapter, not complete. This reference has been updated


**Summary and General Comments**

Reviewer #4: The revised manuscript has largely addressed the points raised by the reviewers, summarises the methodologies applicable to cutoff determination in serological testing and adds insight to the debate on optimal selection of cutoffs for serological bead array-based assays. A missing element in the conclusions are the authors own recommendations for the appropriate methodology in specific settings Box 1 does not add significantly to, or with any more clarity, what is in the results and discussion and appears to be superfluous.

Thank you for the suggestions. The text has been revised with clear recommendations. Box 1 provides a concise summary of findings from the literature review, as well as providing important factors, or guidelines, to refer to when deciding an appropriate cut-off method. Therefore, the value of Box 1 in the main text is worth keeping. To ensure it is clear, we have amended the title to also include the term ‘summary’ and ‘complexities’ to provide a clearer context of Box 1 and have amended the language in the discussion pertaining to Box 1.
--------------------
